# Prior Cocaine Exposure Increases Firing to Immediate Reward While Attenuating Cue and Context Signals Related to Reward Value in the Insula

**DOI:** 10.1523/JNEUROSCI.3025-20.2021

**Published:** 2021-05-26

**Authors:** Heather J. Pribut, Daniela Vázquez, Adam T. Brockett, Alice D. Wei, Stephen S. Tennyson, Matthew R. Roesch

**Affiliations:** ^1^Department of Psychology, University of Maryland, College Park, Maryland 20742; ^2^Program in Neuroscience and Cognitive Science, University of Maryland, College Park, Maryland 20742

**Keywords:** cocaine, context, decision, insula, recording, reward

## Abstract

The insula contributes to behavioral control and is disrupted by substance abuse, yet we know little about the neural signals underlying these functions or how they are disrupted after chronic drug self-administration. Here, male and female rats self-administered either cocaine (experimental group) or sucrose (control) for 12 consecutive days. After a 1 month withdrawal period, we recorded from insula while rats performed a previously learned reward-guided decision-making task. Cocaine-exposed rats were more sensitive to value manipulations and were faster to respond. These behavioral changes were accompanied by elevated counts of neurons in the insula that increased firing to reward. These neurons also fired more strongly at the start of long-delay trials, when a more immediate reward would be expected, and fired less strongly in anticipation of the actual delivery of delayed rewards. Although reward-related firing to immediate reward was enhanced after cocaine self-administration, reward-predicting cue and context signals were attenuated. In addition to revealing novel firing patterns unique to insula, our data suggest changes in such neural activity likely contribute to impaired decision making observed after drug use.

**SIGNIFICANCE STATEMENT** The insula plays a clear role in drug addiction and drug-induced impairments of decision making, yet there is little understanding of its underlying neural signals. We found that chronic cocaine self-administration reduces cue and context encoding in insula while enhancing signals related to immediate reward. These changes in neural activity likely contribute to impaired decision making and impulsivity observed after drug use.

## Introduction

The insula has recently gained traction as a key contributor to relapse and drug-seeking behaviors and as a potential therapeutic target for addiction. Work in humans has shown that the insula is activated during the presentation of drug cues and recollection of past drug use (Wang et al., 1999; Bonson et al., 2002). Further, it has been shown that damage to the insula can promote drug abstinence (Naqvi et al., 2007). Rodent work has supported these ideas by showing that disruption of insula function reduces the ability of drug-associated cues and contexts to drive drug seeking (Contreras et al., 2007; Forget et al., 2010; Perdomo et al., 2012; García et al., 2013; Li et al., 2013; Pelloux et al., 2013; Seif et al., 2013; Pushparaj and Le Foll, 2015; Cofresí et al., 2019; Campbell et al., 2019; Zhang et al., 2019; Gil-Lievana et al., 2020).

Although the insula is clearly involved in drug addiction, it is less clear how it contributes to normal behavioral control or how those mechanisms might be disrupted by chronic drug abuse. Traditionally, insula has been described as an interoceptive center (Damasio et al., 2000; Craig, 2002, 2010; Naqvi and Bechara, 2010), but is also thought to contribute to functions related to reward processing and decision-making (Mesulam and Mufson, 1982; Ongür and Price, 2000; Preuschoff et al., 2008; Burke and Tobler, 2011; Droutman et al., 2015b; Rogers-Carter and Christianson, 2019). Consistent with these functions, recent work has shown that firing in the insula correlates to the anticipation and delivery of both positive and negative outcomes (Guillem et al., 2010; Samuelsen et al., 2012; Kusumoto-Yoshida et al., 2015; Mizoguchi et al., 2015; Jo and Jung, 2016; Moschak et al., 2018; Vincis et al., 2020; Wittmann et al., 2020). Here, we ask whether outcome-related neural correlates in the insula are disrupted by chronic cocaine self-administration in rats performing a reward-guided decision-making task.

**Figure 1.**
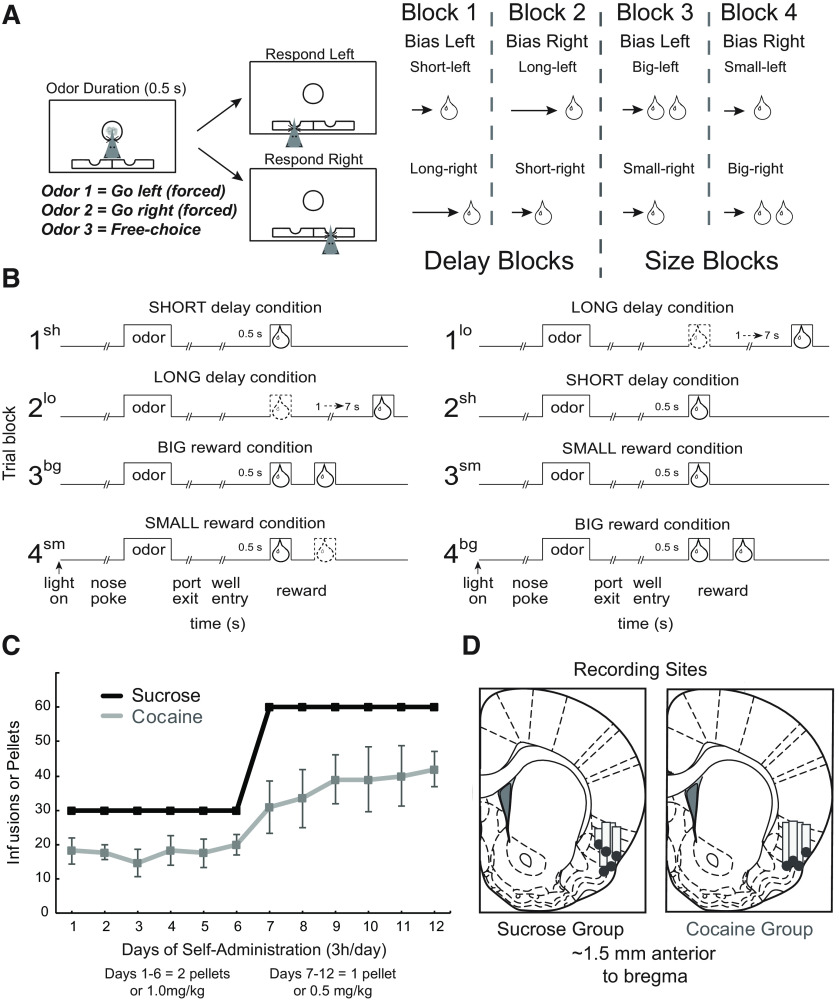
Reward-guided decision-making task, self-administration data, and recording sites. ***A***, Task schematic, showing an example trial (left) and block sequence in a session (right). Rats nose poked in a central port for 0.5 s to receive an odor with a duration of 0.5 s. Odors would instruct them to respond to fluid wells below where they could receive liquid sucrose rewards after 500–7000 ms. ***B***, Summary of block structure. During a recording session, one fluid well would be arbitrarily designated as short (delivering reward after a short 500 ms delay) and the other designated as long (delivering reward after a 1–7 s delay). Contingencies were reversed after ∼60 trials (Block 2). In Block 3, delays to reward were held constant at 500 ms, but the well designated as long in the previous block now offered 2 boli (i.e., big reward), whereas the other well offered only 1 (i.e., small reward). Contingencies switched once again in Block 4. Each block shift was unsignaled so that rats needed to learn through behavior that reward values had changed. ***C***, Average lever-press rates for control (black) and cocaine-exposed (gray) rats for each day of self-administration. Data points represent each day. Bars on the cocaine-exposed group represent SEM. ***D***, Electrode placement for each rat, verified by histology.

During this task (Fig. 1*A,B*) rats use reward-predicting cues to guide choice behavior between two options, the values of which are manipulated across two contexts: in one, rats choose between an immediate and delayed reward; in the other, rats choose between a large and small reward with delays to reward held constant. In both contexts, rats learn which option yields the preferred outcome and maintain those expectations during delays to reward and across trial blocks while following forced-choice rules. We have previously shown that rats prefer immediate over delayed reward, and large over small reward and that rats are more motivated during size block contexts (Roesch et al., 2007; Roesch and Bryden, 2011; Burton et al., 2017, 2018; Vázquez et al., 2019). Further, we have shown that rats that previously self-administered cocaine are more sensitive to value manipulations and exhibit faster reaction times months after drug exposure (Burton et al., 2017, 2018; Brockett et al., 2018; Vázquez et al., 2019).

Given the insula's role in addiction and its dysregulation following drug use, we hypothesized that functional signals related to reward processing would be disrupted by chronic cocaine self-administration. We found reward delivery-related signals in cocaine-exposed rats were enhanced, whereas context- and cue-related firing was reduced. Further, encoding of immediate delivery of reward was more prominent in cocaine-exposed rats. These results suggest that heightened reward responding and lower cue and context selectivity in insula may contribute to increased impulsivity and poor decision making observed after chronic drug use (Roesch et al., 2007; Simon et al., 2007; Mendez et al., 2010; Brockett et al., 2018; Burton et al., 2018; Vázquez et al., 2019).

## Materials and Methods

### 

#### 

##### Subjects

Nine Long–Evans rats (weight, 175–200 g; three females, 6 males) were obtained from Charles River Laboratories. Subjects were held and tested at the University of Maryland, according to university and National Institutes of Health guidelines.

##### Odor-guided delay/size choice task

Rats were trained on the delay/size task (Fig. 1*A,B*) for 1 month before surgery. In brief, subjects needed to nose poke into a central port during house light illumination to receive one of three odor cues (odors were 2-Octanol, Pentyl Acetate, or Carvone). These odors instruct rats in which direction to move to receive a 10% liquid sucrose reward from wells located on either side of the central port. Two of these odors instructed rats to go either left or right for reward (forced choice), whereas the third odor indicated that rats could receive a reward from either well (free choice). Forced-choice odors were counterbalanced across rats and were presented in a pseudorandom sequence with free-choice odor presented on 7/20 trials. Incorrect well selection during a forced-choice trials resulted in no reward delivery.

During each recording session, reward value was independently manipulated across four blocks of 60 correct trials (Fig. 1*A*; Fig. 1*B*). During the first two blocks, one well was randomly designated to deliver reward immediately (500 ms delay, 0.05 ml), whereas the other well delivered sucrose with delays that would gradually increase (1000–7000 ms). Delay contingencies would then be switched at the start of the second block so that the well previously containing higher-valued reward (short delay) now carried the longer delay. During the final two trial blocks, delays on both wells were held constant at 500 ms, and reward value was instead manipulated by size. At the start of the third block, the well that previously carried the long-delay condition now delivered two boli of sucrose solution. Value contingencies were again switched at the fourth block (i.e., well previously containing large reward now contained small reward, and vice versa). Rats were water deprived to increase motivation to complete the task. Water bottles were removed by noon the day before training began. After a session, rats were given water for 20 min. Because testing took place Monday through Friday, rats were given *ad libitum* access to water after Friday's session, and then water bottles were removed Sundays at noon. This deprivation schedule has been used in our previous studies and has been shown to increase motivation in the behavioral task without detriment to the rats' health (Roesch et al., 2007; Roesch and Bryden, 2011; Burton et al., 2017, 2018; Vázquez et al., 2019).

##### Surgery

All subjects were implanted with catheters in the jugular vein for self-administration and drivable electrodes (+1.5 anteroposterior, +/−5.0 mediolateral, −5.0 dorsoventral from brain surface) for single-unit recordings (control = 5, cocaine = 4). Catheters were made from Silastic tubing (0.02 × 0.037 inch, Dow Corning) with a modified G 5-up cannula (Plastics One). The cannula traveled through the fascia layer over the shoulder and was cemented on top of the skull. Electrodes (bundles of 10–25 µm diameter FeNiCr wire) were implanted unilaterally during this same surgery (three left, six right).

##### Self-Administration

After 1 week of recovery, rats underwent a 12 d self-administration protocol in operant chambers (Med Associates). Animals in the cocaine group could press a lever for an infusion of cocaine, whereas control animals pressed for sucrose pellets. Cocaine dosage was calculated by weight, with separate doses calculated for males and females to account for weight differences. On days 1–6, rats self-administered 1 mg/kg dosage of cocaine (experimental group) or 2 sucrose pellets (control) per lever press for a maximum of 30 infusions or for a 3 h time limit. For the final 6 d, the dosage of cocaine was halved to 0.5 mg/kg, and only 1 sucrose pellet was delivered per lever press, for a maximum of 60 presses. This procedure allowed us to assess increases in drug seeking when doses are cut in half to maintain the desired level of drug intake.

Importantly, rats were trained on the delay/size task before cocaine self-administration occurred. No behavioral or recording data were collected during cocaine exposure or during the 1 month withdrawal period. Self-administration procedures expose rats to cocaine before recording during the delay/size task to determine how drug exposure affects brain and behavior in the long term. These procedures are consistent with work establishing that continuous access to high cocaine doses evokes drug-taking and drug-seeking behaviors that are consistent with promoting symptoms of addiction (Allain and Samaha, 2019) and have been shown to change behavior and neural signals in other brain regions (Calu et al., 2007; Burton et al., 2017, 2018; Brockett et al., 2018; Vázquez et al., 2019).

##### Single-unit recording

Procedures were the same as described previously (Bryden and Roesch, 2015). Wires were screened for activity each day; if no activity was detected, the rat was removed, and the electrode assembly was advanced 40 or 80 µm. Otherwise, a session was conducted, and the electrode was advanced at the end of the session. Neural activity was recorded using four identical Plexon Multichannel Acquisition Processor systems. Signals from electrode wires were amplified 20× by an op-amp headstage located on the electrode array. Immediately outside the training chamber, the signals were passed through a differential preamplifier (Plexon, PBX2/16sp-r-G50/16fp-G50) where single unit signals were amplified 50× and filtered at 150-9000 Hz. The single unit signals were then sent to the Multichannel Acquisition Processor box, where they were further filtered at 250-8000 Hz, digitized at 40 kHz, and amplified at 1–32×. Waveforms (>2.5:1 signal-to-noise) were extracted from active channels and recorded to the disk by an associated workstation with event time stamps from the behavior computer.

##### Experimental design and statistical analyses

Behavior in the recording task was analyzed by calculating the percentage of correct responses on forced-choice trials (i.e., the amount of trials the animal correctly responded to the side corresponding to the directional odor cue), the percentage of trials in which rats chose a particular valued condition (short, long, large, small) on free-choice trials, and reaction times (odor offset to odor port exit). These calculations included all trials in their respective categories, including those following a block switch. Behavioral analyses were computed for each individual session (separated by cocaine and control groups) and then averaged across sessions for each group. Conducting analyses across sessions—instead of across individual subjects—provides a better reflection of the neural correlates corresponding to behavior. Importantly, the main behavioral findings described in this article have been replicated in three different studies (Burton et al., 2017, 2018; Vázquez et al., 2019). Multifactor analysis of variance factors included group (control vs cocaine), reward value (high vs low), and value manipulation (size vs delay). *Post hoc* between-subjects *t* tests (*p* < 0.05) were used to determine differences between the cocaine and control groups.

Single units were analyzed in Offline Sorter (Plexon) using template-matching software and were exported to NeuroExplorer (Nex Technologies) and MATLAB (MathWorks). Again, all rewarded trials were analyzed, from both free and forced-choice trials, including trials that immediately followed a block switch. Neural activity was analyzed in three epochs: baseline firing rate, odor cue onset, and reward onset. All were calculated by dividing the total number of spikes by time. Baseline activity was taken 1 s before odor presentation, the odor epoch was taken 100 ms after odor onset until port exit, and the reward epoch was taken 250 ms before sucrose delivery to 1 s after reward delivery. This epoch was designed to capture activity related to reward expectancy and delivery. Importantly, there was no overlap between epochs, even during short delay (500 ms) conditions. To analyze neural activity during these epochs, we normalized firing rates so that high- and low-firing cells could be analyzed together. We determined significant changes in firing rates by taking difference scores for each neuron and plotting the number of neurons with significant differential firing from baseline (demonstrated through Wilcoxon tests, *p* < 0.05). Relationships between neural firing and behavioral activity were determined using regression analyses.

##### Histology

Upon completion of recordings, subjects were deeply anesthetized via isoflurane and perfused transcardially with 200 ml of 0.9% saline, followed by 500 ml of 4% paraformaldehyde (PFA). Brains were held in 4% PFA until ready for slicing, at which time they were placed in a 20% sucrose in PBS until the brains had sunk. We then sectioned brains at 50 µm and used a Cressyl violet stain to confirm electrode placement (Fig. 1*D*).

##### Data availability

Raw data and MATLAB codes used for behavioral and neural analyses available on request.

## Results

### Cocaine exposure produced a response bias toward high-valued rewards

Behavioral data from 416 cocaine recording sessions (for each rat, *n* = 128, 115, 89, 84,) and 315 control sessions (*n* = 81, 75, 74, 72, 13; Fig. 1*D*) were analyzed using a multifactor ANOVA across dependent variables of percentage choice, percentage correct, and reaction times on forced- and free-choice trials. The ANOVA factors consisted of group (control vs cocaine), value (high vs low), and block (delay vs size).

**Figure 2.**
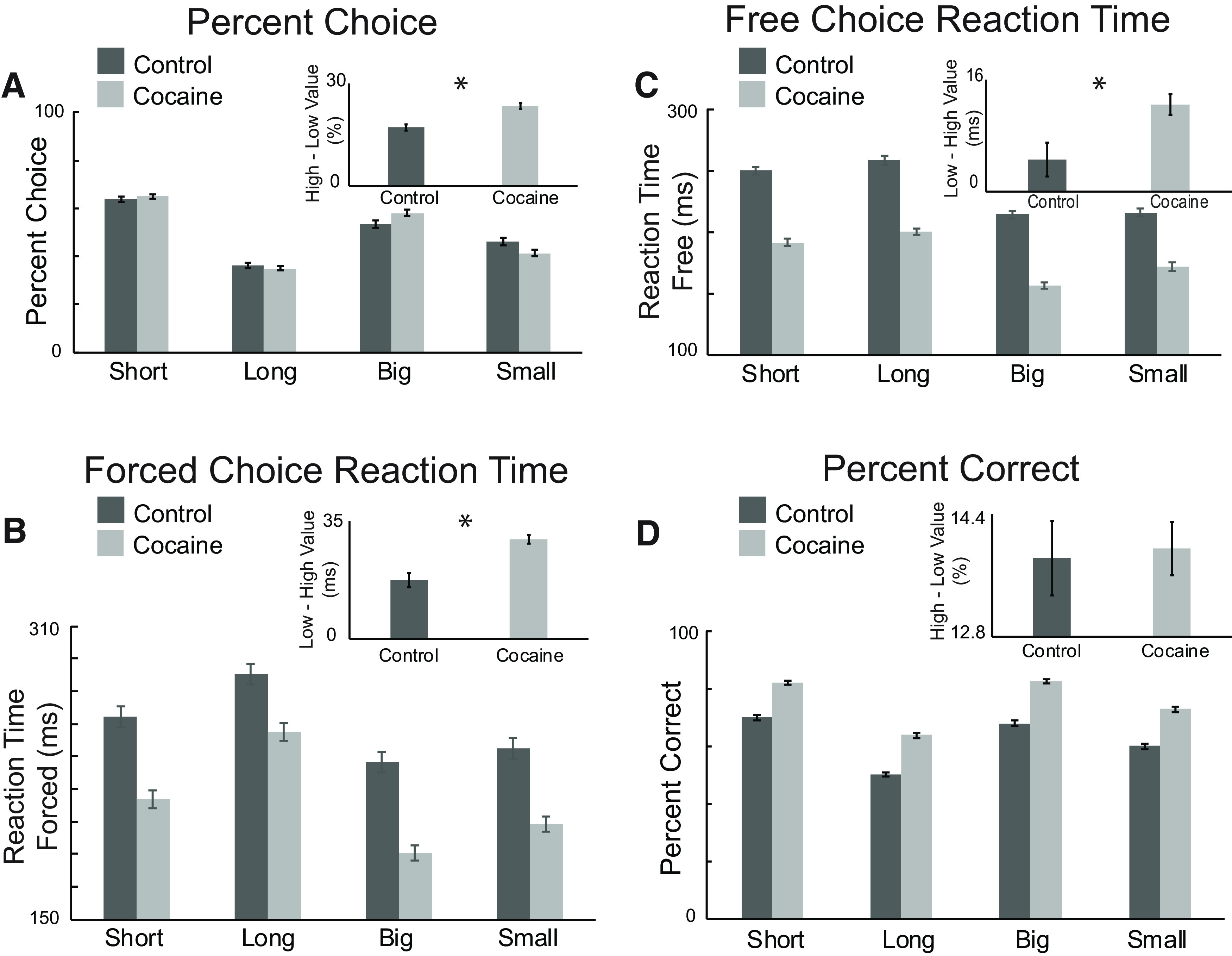
Cocaine exposure produced faster reaction times and response biases for high-valued rewards. ***A***, Percent choice on all free-choice trials in each value manipulation. Results were averaged across animals and weighted by the number of recordings performed to provide a better representation of the behaviors observed as related to the neural analysis (controls: dark bars; cocaine: light bars). Inset, Response bias (percent choice high- to low-value rewards). Asterisk signifies *p* < 0.05. ***B***, Reaction time (odor port exit–odor onset) on forced-choice trials for each value manipulation. Inset, Response bias (reaction time low- to high-value rewards). Asterisk signifies *p* < 0.05.. ***C***, Reaction time for free-choice trials. Inset, Response bias, calculated as in ***C***. Asterisk signifies *p* < 0.05. ***D***, Percentage correct on all forced-choice trials. Inset, Response bias, calculated as in ***A***. All error bars indicate SEM.

Analyzing free-choice trials (Fig. 2*A*) found a significant main effect of value (*F*_(1,5841)_ = 1077.58, *p* < 0.001, ANOVA); selection of high value rewards was significantly greater in both delay (*t*_(1461)_ = −53.458, *p* < 0.001, unpaired *t* test) and size blocks (*t*_(1461)_ = −21.551, *p* < 0.001, unpaired *t* test). Cocaine rats' stronger preference for high-value rewards is further illustrated (Fig. 2*A*, inset) in the difference between high (i.e., short, big) and low (i.e., long, small) value free-choice responding for control and cocaine-exposed rats (*t*_(707)_ = −5.026, *p* < 0.001, unpaired *t* test).

The exaggerated response bias in cocaine rats toward high-valued reward was also observed in reaction times. Main effects of value and block during forced-choice trials demonstrated that all rats were faster for high-valued rewards (Fig. 2*B*; *F*_(1,5841)_ = 69.26, *p* < 0.001, ANOVA), and were faster for size compared with delay blocks (*F*_(1,5841)_ = 209.49, *p* <0.001, ANOVA). There was also a main effect of cocaine, indicating that rats that had self-administered cocaine were altogether faster compared with controls (*F*_(1,5841)_ = 276.87, *p* < 0.001, ANOVA). Finally, cocaine-exposed rats showed a stronger response bias for high- versus low-value rewards (Fig. 2*B*, inset; significant interaction between group and value, *F*_(1,5841_) = 4.7, *p* = 0.030, ANOVA; *t*_(543)_ = 4.856, *p* < 0.001, unpaired *t* test). Thus, cocaine-exposed rats exhibited an exaggerated response bias toward high-valued rewards, as well as faster reaction times.

Similarly, reaction times during free-choice trials (Fig. 2*C*) were faster for high-valued rewards (main effect of value, *F*_(1,5717)_ = 11.05, *p* < 0.001, ANOVA) and during size blocks compared with delay blocks (main effect of block, *F*_(1,5717)_ = 186.14, *p* < 0.001, ANOVA). Rats that self-administered cocaine were also significantly faster compared with controls overall (main effect of group, *F*_(1,5841)_ = 443.5, *p* < 0.001, ANOVA), particularly for high-valued rewards (Fig. 2*C*, inset; *t*_(537)_ = 2.733, *p* = 0.006, unpaired *t* test).

Altogether, these results replicate previous findings that rats demonstrate a preference for high-valued rewards through their choices and reaction times and that responding is biased toward high-valued rewards in animals previously exposed to cocaine (Burton et al., 2017, 2018; Brockett et al., 2018; Vázquez et al., 2019). Interestingly, in contrast to previous studies, we found that cocaine-exposed rats were overall more accurate on forced-choice trials (Fig. 2*D*). There were significant main effects of block and value, with rats being more accurate during size blocks (*F*_(1,5841)_ = 90.09, *p* < 0.001, ANOVA) and for high-valued rewards (*F*_(1,5841)_ = 945.27, *p* < 0.001, ANOVA). An additional main effect of group showed cocaine rats altogether followed forced-choice rules more accurately compared with controls (*F*_(1,5841)_ = 866.17, *p* < 0.001, ANOVA), more strongly adhering to stimulus-response contingencies (i.e., odor 1 = go left; odor 2 = go right).

### Cue-related value encoding was attenuated after cocaine exposure

**Figure 3.**
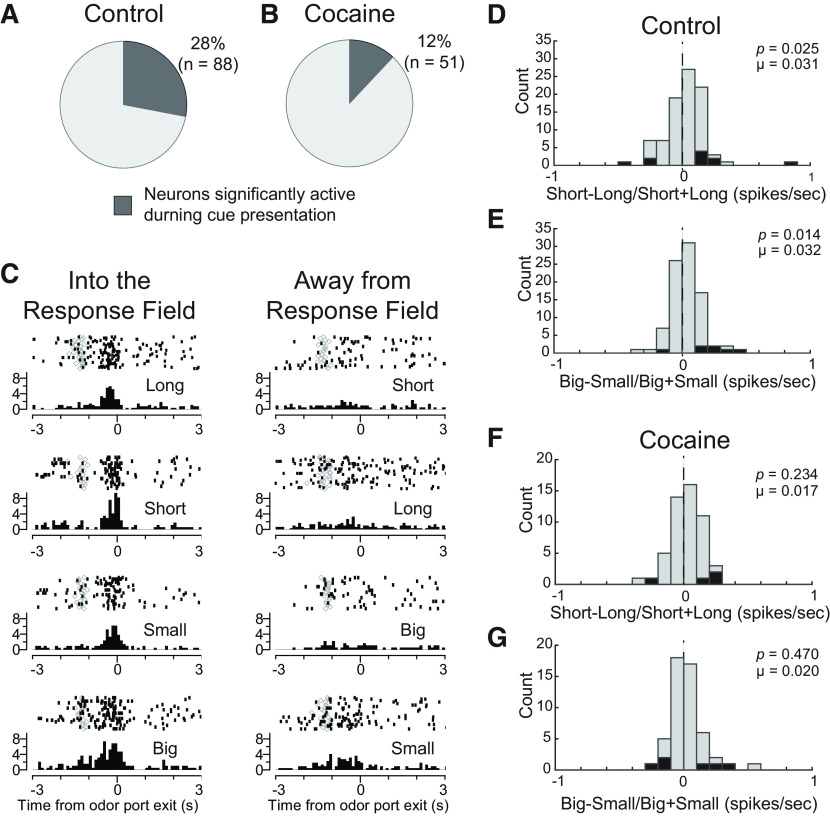
Cocaine exposure reduced cue-induced activity and value encoding from odor-response cells. Selective versus nonselective odor-responsive cells from control (***A***) and cocaine-exposed (***B***) rats. Selectivity was defined by significantly greater firing rates during the odor analysis epoch (100 ms after odor onset to odor port exit) compared with the baseline analysis epoch (1 s before odor onset; Wilcoxon, *p* < 0.05). ***C***, Raster plots from an odor-responsive example cell. Activity is aligned to odor unpoke (i.e., when the rat leaves the odor port), with each row displaying activity for each trial type. ***D***, Distribution of value analysis (short–long / short + long; big–small/big + small) for cells taken from control rats, comparing firing rates for short- and long-delay rewards. ***E***, Similar analysis as ***D***, comparing firing rates for big and small rewards during blocks where reward size is manipulated. ***F*, *G***, Same analyses as ***D***, ***E***, but for cells from cocaine-exposed rats. Black bars represent counts of neurons with firing that significantly differed between trial types (*p* < 0.05, Wilcoxon).

We first asked whether cocaine exposure altered the counts of insula neurons that were responsive during odor sampling (odor epoch: 100 ms after odor onset to port exit) compared with baseline (1 s before odor onset; *p* < 0.05, Wilcoxon). In controls, 28% of neurons (*n* = 88/315 cells) were responsive during odor sampling (Fig. 3*A*), whereas only 12% (*n* =51/416 cells) were responsive in rats that self-administered cocaine (Fig. 3*B*). Both counts were significantly greater than expected by chance alone (control: χ^2^ = 617.371, *p* < 0.001, χ^2^; cocaine: χ^2^ = 135.51, *p* < 0.001, χ^2^), and the frequency of cells that increased firing in controls was significantly higher compared with cells from cocaine-exposed rats (χ^2^ = 18.395, *p* < 0.001, χ^2^). Thus, rats that had self-administered cocaine had fewer cue-responsive neurons compared with controls. An example of a cue-responsive neuron is illustrated in Figure 3*C*. This neuron fired more strongly for odor cues that predicted higher value reward for behavioral responses to be made into the cell's response field (i.e., the cell's preferred response direction or the direction that elicited the strongest firing; Fig. 3*C*, left).

To quantify value selectivity during the odor epoch, we computed the difference between firing for high- and low-valued rewards in each neuron during this epoch (i.e., odor onset to port exit) for delay (delay index = short – long / short + long) and size blocks (size index = big – small / big + small), for responses made into each cell's response field. For controls, there were significant positive shifts in delay (*p* = 0.025, µ = 0.031, Wilcoxon; Fig. 3*D*) and size (*p* = 0.014, µ = 0.032, Wilcoxon; Fig. 3*E*) indices, indicating a higher frequency of neurons with stronger firing rates to cues that predicted high-valued reward within size and delay trial blocks (black bars represent counts of neurons with firing that significantly differed between trial types; *p* < 0.05, Wilcoxon). In rats that self-administered cocaine, there were no significant shifts in either delay (*p* = 0.234, µ = 0.017, Wilcoxon; Fig. 3*F*) or size indices (*p* = 0.470, µ = 0.020, Wilcoxon; Fig. 3*G*); however, neither were significantly different from control distributions (delay: *z* = −0.754, *p* = 0.451; size: *z* = −0.889, *p* = 0.374, Wilcoxon). Thus, we conclude that cocaine self-administration diminished value encoding during odor sampling by reducing the counts of odor-responsive neurons (Fig. 3*A* vs Fig. 3*B*), and only slightly attenuating outcome selectivity in those that remained (Fig. 3*D,E* vs Fig. 3*F,G*).

### Immediate rewards were more strongly represented after cocaine exposure

**Figure 4.**
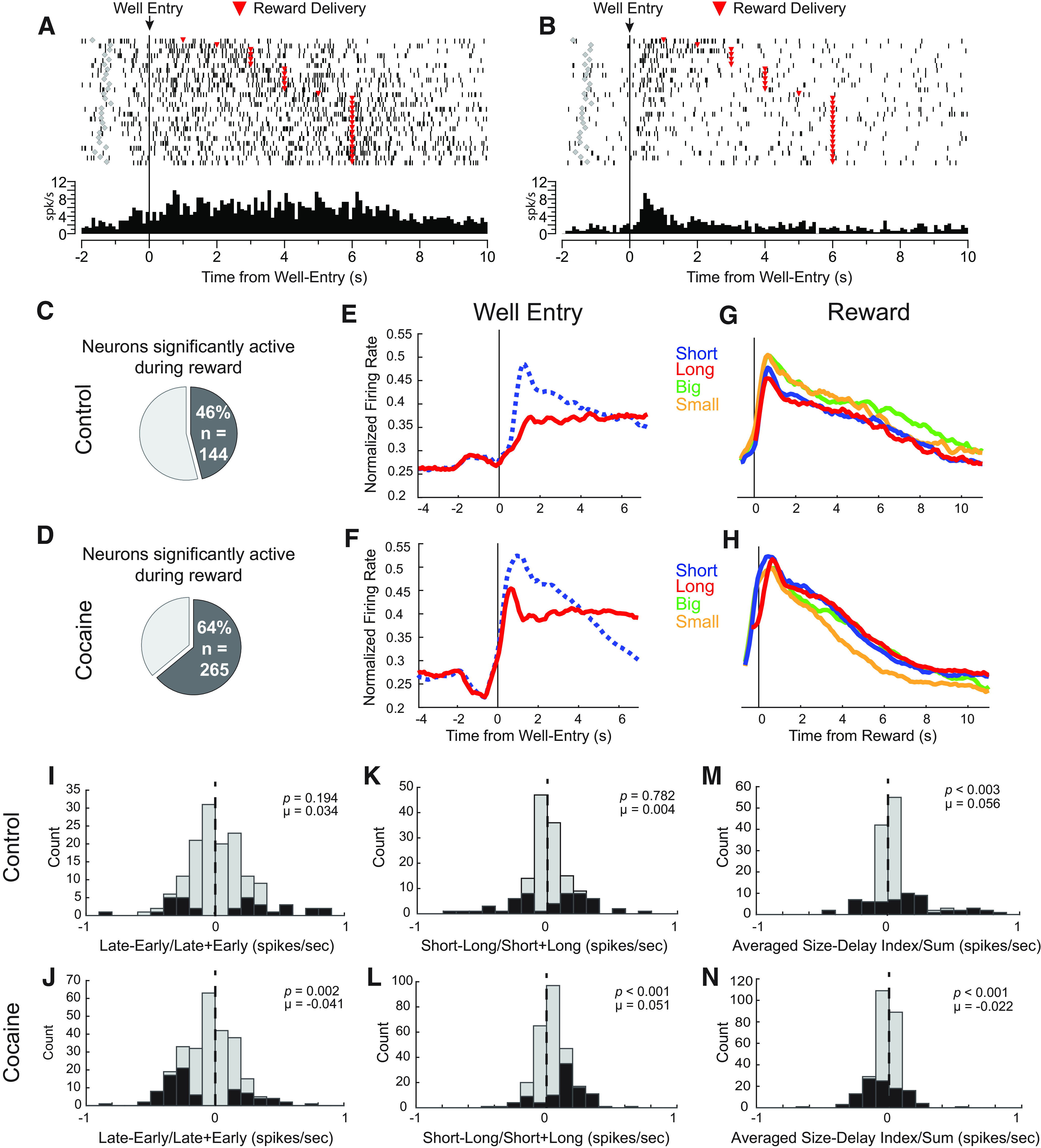
Cocaine exposure reduced delay representation, and activity was discounted for long-delay rewards in reward-responsive cells. ***A***, Single neuron example during a long-delay trial in which activity is sustained during the delay period. Activity is aligned to well entry (time 0, black line). ***B***, Single neuron example during a long-delay trial in which activity initially increases after well entry and subsequently decreases, failing to maintain reward representation throughout the long delay. ***C***, ***D***, Percentage of reward-selective cells from control (***C***) and cocaine-exposed (***D***) rats, as defined by significantly greater firing during the reward analysis epoch (250 ms before reward to 1 s after reward delivery) compared with baseline (1 s before odor onset; Wilcoxon, *p* < 0.05). ***E***, ***F***, Normalized firing rates for neurons that increased firing rates during the reward epoch from control (*n* = 144; ***E***) and cocaine-exposed rats (*n* = 265; ***F***). Activity is aligned to fluid well entry, comparing short (blue dotted line) and long-delay (red line) rewards. ***G***, ***H***, Normalized firing rates for cells from control (***G***) and cocaine-exposed (***H***) rats, aligning activity for short (blue), long (red), big (green), and small (orange) rewards to reward delivery. ***I***, ***J***, Distribution of value indices for cells taken from control (***I***) and cocaine-exposed (***J***) rats, comparing firing rates during the first and last 500 ms of long delay trials. ***K***, ***L***, Distribution of delay indices (short – long / short + long) for cells taken from control (***K***) and cocaine-exposed (***L***) rats, comparing firing rates during the reward epoch. ***M***, ***N***, Distribution of context indices reflecting changes in global value (size block – delay block / size block + delay block) comparing average activity during size and delay blocks during the postreward delivery epoch (1–2 s after reward delivery), for control (***M***) and cocaine-exposed (***N***) rats. Black bars represent counts of neurons with firing that significantly differed between trial types (*p* < 0.05, Wilcoxon).

Our next analyses examined neurons whose activity increased during the anticipation and delivery of reward. In controls, 46% (*n* = 144) of neurons increased firing during the reward epoch (Fig. 4*C*; 250 ms before reward delivery to 1 s after reward delivery) compared with baseline (Wilcoxon test, *p* < 0.05; χ^2^ test = 1568.594, *p* < 0.001). In rats that self-administered cocaine, we found an 18% increase in the counts of reward-responsive neurons (Fig. 4*D*; 64%, *n* = 265). The proportion of reward-responsive neurons in cocaine-exposed rats was significantly greater than the proportion observed in controls (χ*^2^* = 6.454, *p* = 0.011, χ^2^). Single-cell examples from this neuronal population are illustrated in Figure 4*A* and *B*. Many neurons fired in anticipation of reward across long delays, exhibiting sustained firing from well entry until reward delivery (Fig. 4*A*). However, other neurons seemed to fail in representing reward across a long delay (Fig. 4*B*). These cells exhibited increases in firing after well entry at the time when reward would have been delivered on the majority of trials (i.e., after 500 ms), followed by a decrease in firing during the remainder of the delay. To determine response patterns across the entire population of reward responsive neurons, we plotted average firing aligned to both well entry (Fig. 4*E,F*) and reward delivery (Fig. 4*G,H*) for actions made into the response field.

Aligning activity to well entry, we found that insula neurons from control rats exhibited sustained elevated firing during long delays, starting at well entry (Fig. 4*E*, red). In controls, anticipatory firing after well entry for longer delays (red) rose less rapidly than for rewards delivered after a short delay (blue dashed). This was not true after cocaine exposure (Fig. 4*F*). Instead, early activity during long delays tracked firing as if on a short-delay trial, peaking around the time when the more immediate reward would have been delivered before dropping to sustained levels. Thus, qualitatively it appeared that firing after cocaine exposure held onto the expectation that reward would be delivered after a shorter delay, which occurs on three (short, big, small) of the four trial types).

To quantify this observation, we computed the difference in activity during the first and last 500 ms of the delay period on long-delay trials (late – early / late + early; early = first 500 ms after well entry; late = last 500 ms before reward). These two time points encompass nonoverlapping activity early and late in the delay period, when rats stay in the fluid well before reward delivery. In controls, we observed no significant shift in the distribution, demonstrating that both early and late expectancy-related firing were similarly represented across longer delays (*p* = 0.194, µ = 0.034, Wilcoxon; Fig. 4*I*). However, for rats that self-administered cocaine, this distribution was significantly shifted below 0 (*p* = 0.002, µ = 0.041, Wilcoxon; Fig. 4*J*) and significantly different from the control distribution (*z* = −2.866, *p* = 0.004; Wilcoxon), indicating that cocaine-exposed rats had a higher frequency of neurons firing more strongly at the start compared with the end of long delays.

These results indicate that in cocaine-exposed rats, neural correlates of immediate reward expectancy were maintained during performance of long-delay trials. These findings also suggest that cocaine exposure impairs the ability of insula neurons to maintain expectancy-related firing from early to late in delay trials. Such a lack of firing might lead to a reduced or discounted neural representation of delayed rewards compared with those delivered after 500 ms (i.e., short-delay trials). Overall, this result suggests that immediate rewards are better represented in the insula after cocaine exposure. Indeed, examining average firing aligned to reward delivery (Fig. 4*G,H*) suggests that anticipatory firing for immediate rewards (i.e., short-delay trials, Fig. 4*H*, blue) was stronger compared with firing for delayed rewards (Fig. 4*H*, red). To quantify this effect, we computed the delay indices (short − long / short + long) for each neuron during the reward epoch. The distribution of delay indices from rats that self-administered cocaine were significantly shifted in the positive direction (*p* < 0.001, µ = 0.051, Wilcoxon; Fig. 4*L*) and significantly different from the control distribution (*p* = 0.782, µ = 0.004, Wilcoxon; Fig. 4*K*; cocaine vs control *z* = 3.081, *p* = 0.002, Wilcoxon).

These results suggest that previous cocaine exposure increased neural signals for immediate rewards during short-delay trials and also overrepresented the anticipation of more immediate reward, both within long-delay trials themselves and when directly comparing immediate to delayed reward at the time of delivery.

### Context-related value signals were attenuated by cocaine exposure

On constructing the population histograms for the analysis above (Fig. 4*G,H*), we unexpectedly found that neurons in the insula appeared to encode block context in control rats. Specifically, firing was higher after reward delivery during size blocks (green and orange vs blue and red). Remarkably, this was true even for small rewards (orange) that were physically the same delay and size as rewards on short-delay trials (blue).

To quantify this effect, we created a context index comparing average activity from size and delay blocks (size block – delay block / size block + delay block) 1–2 s after reward that indexed the global value differences between blocks (Fig. 4*M,N*). This analysis epoch occurs after completion of the trial, while rats are still consuming rewards and are aware of the reward that was just delivered. We found insula neurons exhibited a positive shift in distributions of activity, confirming this unexpected novel finding of higher firing after reward delivery during size compared with delay blocks at the level of single neurons (*p* = 0.003, µ = 0.056, Wilcoxon; Fig. 4*M*). Interestingly, this effect was not observed in cocaine-exposed rats. Instead, the context index distribution was significantly shifted below zero, indicating that cells tended to fire more strongly for delay blocks compared with size blocks (*p* = 0.001, µ = −0.022, Wilcoxon; Fig. 4*N*; cocaine vs control: *z* = −4.287, *p* < 0.001, Wilcoxon).

**Figure 5.**
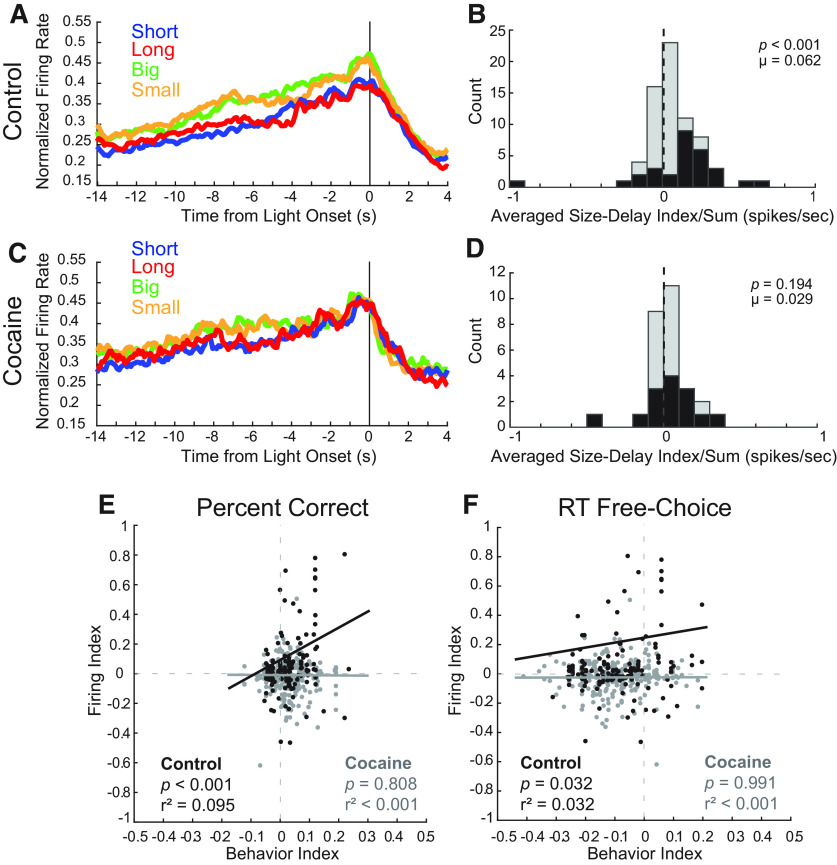
Cocaine exposure reduced context encoding and its relationships with behavior. ***A***, Normalized firing rates for cells from control rats that exhibited elevated activity before trial onset. ***B***, Distribution of context indices for controls (*n* = 69) reflecting changes in global value (size block – delay block / size block + delay block) comparing average activity during size and delay blocks during the pretrial epoch (4 s before light onset). Black bars represent counts of neurons with firing that significantly differed between trial types (*p* < 0.05, Wilcoxon). ***C***, ***D***, Same as ***A*** and ***B*** for cocaine-exposed rats (*n* = 28). ***E***, ***F***, Scatter plots of context index (size block – delay block / size block + delay block) distribution data from neurons that increased activity during the reward epoch compared with ratios of size versus delay behavior for percent correct (***E***), **f**ree-choice reaction times (***F***). Black = control, gray = cocaine. RT, Reaction times.

Remarkably, we also observed that neurons that ramped up firing in anticipation of house light onset (i.e., the stimulus that signaled the start of each trial) fired more strongly during size compared with delay trial blocks. This finding is illustrated in Figure 5*A*, which plots the average firing of 69 control neurons (22% of total). To quantify this effect, we again computed the context index during the 4 s before light onset (the period of time immediately preceding the onset of the trial; Fig. 5*B*). Consistent with observations in the population histogram, we found a significant positive shift in firing rate distributions, demonstrating that insula neurons tended to fire more strongly during size compared with delay blocks before trial onset (*p* < 0.001, µ = 0.062, Wilcoxon).

Interestingly, cells that carried this signal were nearly nonexistent in rats that had self-administered cocaine (Fig. 5*C*). Only 7% (*n* = 28) of neurons exhibited such pretrial ramping activity, which were significantly fewer cells compared with such neurons found in controls (χ*^2^* = 25.963, *p* < 0.001, χ*^2^*). For those cells, the context index distribution was not significantly shifted (*p* = 0.194, µ = 0.029, Wilcoxon; Fig. 5*D*), but was not significantly different compared with controls (*z* = −1.071, *p* = 0.284, Wilcoxon). Thus, we conclude that cocaine self-administration diminished contextual pretrial value encoding by reducing counts of responsive neurons and slightly attenuating selectivity in those that remained.

Notably, we have not observed these context signals in any other brain area that we have recorded from in rats performing this task (Roesch et al., 2006, 2007, 2012; Bryden et al., 2011; Roesch and Bryden, 2011; Kashtelyan et al., 2012; Burton et al., 2014a,b, 2015, 2017, 2018; Takahashi et al., 2017; Brockett et al., 2018; Vázquez et al., 2019). To better understand the relationship between context-related firing and behavior, we correlated differences in firing between size and delay blocks to differences in percentage corrects and reaction times (size – delay / size + delay).

In control rats, there was a significant positive relationship between size versus delay block accuracy and context encoding (*p* < 0.001, *r*^2^ = 0.095, regression). Thus, increases in firing were correlated with better performance. Interestingly, after cocaine exposure there was no correlation between firing and percentage correct (*p* = 0.808, *r*^2^ < 0.001, regression; Fig. 5*E*). During free-choice trials there was also a significant, positive relationship between firing rate and reaction time in control rats (*p* = 0.032, *r*^2^ = 0.032, regression). This result suggests that stronger firing rates were actually related to slower reaction times during size blocks. This relationship was also disrupted by cocaine exposure (*p* = 0.991, *r*^2^ < 0.001, regression; Fig. 5*F*). Together, these novel firing patterns in the insula are related to accuracy and reaction time during our delay/size task. These relationships are additionally disrupted by cocaine exposure.

## Discussion

The insula's traditional roles integrating external experiences with internal state have more recently expanded to reward-guided decision making (Droutman et al., 2015a). Previous recording studies demonstrate insula activity encodes anticipation of both positive (Samuelsen et al., 2012, 2013) and negative consequences (Jo and Jung, 2016) and reward consumption (Kusumoto-Yoshida et al., 2015; Wittmann et al., 2020). The insula also seems to track the subjective value of reward, reflecting how context and experience change a reward's value even when the characteristics of the reward itself has not changed. Satiety, for example, reduces insula activity for food (Livneh et al., 2017) and water (Livneh et al., 2020; Namboodiri and Stuber, 2020). Other work has shown that when a previously appetitive sucrose solution is paired with delayed (instead of immediate) access to cocaine, insula activity reflects the reduced preference of this once favorable reward, even when the sucrose itself is unchanged (Moschak et al., 2018). Our work demonstrates signals in the insula related to cues, context, and differently valued rewards and how chronic cocaine profoundly affects insula activity in reward-guided decision making. First, we see enhanced signals representing reward, especially delivery-related processing of immediate reward. Second, cocaine exposure reduces signals related to cue and block context selectivity, suggesting the insula plays an important part in drug-induced impairments of executive control and decision making.

Specifically, our study found that the insula encoded predicted outcomes during presentation of reward-predictive odor cues and fired in anticipation of reward after completion of an instrumental response. Further, we showed firing was higher for cues that predicted high-valued reward in both delay and size domains. These neurons likely contributed to subjective preferences of immediate reward and maintenance of reward representations across delays. Interestingly, these firing patterns are similar to what we have previously reported in the orbitofrontal cortex and the nucleus accumbens (Takahashi et al., 2009; Burton et al., 2018). Both regions are outcome-selective during cues and fire during the anticipation and delivery of rewards. However, the insula's firing did not differ between rewards delivered after short and long delays in control rats. Thus, in normal insula, anticipatory reward signals were not discounted by the length of the delay that preceded it.

Interestingly, we also saw that average firing was stronger during size blocks and correlated with changes in reaction time and accuracy. When designing our task, our goal was to completely counterbalance value manipulation and direction within each session. We accomplished this objective by running size blocks last, countering satiation by promising rats more and faster access to reward later in the task. Indeed, rats exhibited faster reaction times and better percentage correct scores during size blocks because of the increased benefit of completing trials (i.e., no long delays and potential for a larger reward). These distinct differences in behavior indicate changes in overall value between delay and size blocks, which may trigger increases in contextual firing observed in the present study. This interpretation is further supported when considering that higher firing during size blocks reflects more than just receiving a larger reward within trials, as activity was stronger before trial initiation, and after delivery of small rewards that were physically identical to short-delay rewards previously experienced during delay blocks (i.e., both trial types delivered 1 bolus after 500 ms delay). However, presenting these size blocks last raises the question of whether this context encoding may be a reflection of satiety or a recency-weighted response to blocks in the second half of the task. Directly testing this issue has proven difficult in the past as we have found that rats are highly unmotivated to work for delayed rewards when presented with size blocks first. Although this is beyond the scope of the current study, readdressing this question—perhaps through shorter recording sessions with reversed delay and size blocks—may add interesting insight to these data.

It may therefore be more accurate to hypothesize that these increases in activity track overall value of block contexts and contribute to elevating motivation as satiation sets in, based on insula activity's correlations to reaction time and percentage correct. Such interpretations are in line with Wittmann et al., (2020), which implicates the macaque insula in the tracking of long-term reward by demonstrating a relationship between BOLD signaling and task engagement during blocks of trials where probability of reward receipt is overall either higher or lower. Our context effects suggest such global reward signals in the insula are encoded at the single-cell level and may represent specific reward outcomes in addition to reward probability. Moreover, our work supports long-held theories of the insula's interoceptive functions and demonstrates novel context-based adjustments to representations of reward value to adjust behavior (Naqvi and Bechara, 2010; Droutman et al., 2015b).

We also determined how these correlates are disrupted after chronic cocaine self-administration. Our results replicate previous behavioral findings that preference for high-valued rewards is exaggerated in cocaine-exposed animals (Roesch et al., 2007; Roesch and Bryden, 2011; Burton et al., 2017, 2018; Vázquez et al., 2019). Interestingly, prior cocaine self-administration also shifted the balance of encoding in insula from cues and context to reward. Specifically, in rats that self-administered cocaine, fewer neurons increased firing to cues and context. Instead, significantly more neurons increased firing in anticipation of immediate reward. This activity might encourage impulsive choice by not adequately conveying the value of differently delayed and sized rewards at the time of the decision and by more strongly representing the anticipation that reward should have been delivered sooner. Consistent with this hypothesis, not only did insula neurons fire more strongly in anticipation of reward on short-delay trials but they also fired strongly at the start of long-delay trials at the time when reward would have been delivered on trials with shorter delays (i.e., 500 ms after well entry).

Remarkably, insula neurons in cocaine-exposed rats also failed to increase firing at the end of recording sessions during size trial blocks. Above, we suggest this signal is important for motivating satiated animals when they encounter global increases in reward value in the context of size blocks. The explanation for its absence in cocaine-exposed rats may be that the signals (related to satiety, motivation, or absolute value) necessary to drive behavior during size blocks are disrupted after cocaine exposure. Alternatively, these size-encoding signals may be absent because they are not necessary to drive motivation in cocaine-exposed rats, as seen by their faster reaction times and higher accuracy in size blocks. However, the absence of these global signals may in turn disrupt processing of long- versus short-delay rewards and subsequently bias behavior for immediate rewards.

Relatedly, we found cocaine exposure disrupted relationships between context encoding and behavior, specifically, greater accuracy and slower reaction time were both associated with higher rates of context encoding. The implications of these relationships appear counterintuitive at face value, especially given that control and cocaine-exposed rats exhibited faster reaction times during size blocks. These correlations may reflect subtle increases in deliberation and attention necessary for performance as fatigue or satiety sets in, especially given the relationship between the insula and the anterior cingulate cortex (ACC) in governing conflict and attention (Damasio et al., 2000; Corbetta and Shulman, 2002; Seeley et al., 2007; Cai et al., 2014; Goulden et al., 2014; Arulpragasam et al., 2018; Porter et al., 2020). Our lab has previously found attentional signals in the ACC and behavior to be disrupted after cocaine exposure (Vázquez et al., 2019). Exploring this hypothesis may therefore be a promising area of future work. The proposed reduction of context and task structure representations in favor of reward additionally fits well with theories of drug exposure enhancing model-free behavior (Jentsch and Taylor, 1999; Volkow and Fowler, 2000; Everitt and Robbins, 2005; Dayan and Berridge, 2014; Lucantonio et al., 2014) and could be explored further by examining insula activity in behavioral tasks where selection of immediate reward is not always the most favorable action.

Finally, we must note that insula spans a large portion of the brain, and across humans, primates, and rodents, its function varies depending on subregion (Naqvi and Bechara, 2009; Droutman et al., 2015b; Gogolla, 2017). We examined a portion of the anterior insula, which itself can be split into dorsal and ventral subregions. As a whole, the anterior insula can be associated with cognitive function (Gogolla, 2017), which was relevant for the present study. Human and primate studies suggest this region can be further differentiated, with the ventral portion responsible for social-emotional processing and the dorsal region responsible for cognition (Droutman et al., 2015a). To our knowledge these same distinctions have not been found in the rodent brain, but greater understanding of the insula's anatomy will be important for instructing future studies.

Thus, our findings demonstrate that cocaine exposure enhances signals related to reward delivery, whereas attenuating signals related to cue and context. Our novel context encoding results provide physiological evidence of the insula's role in interoception and decision making (Naqvi and Bechara, 2010). Moreover, disruptions of these signals and related changes in behavior because of cocaine exposure suggest an important role for the insula in impaired decision making observed after drug abuse. Further study of the insula using different behavioral assays will be important for understanding how this region is involved in both optimal decision making and its drug-induced impairments.
